# Digital health literacy and well-being in university students in Germany: Kevin Dadaczynski

**DOI:** 10.1093/eurpub/ckac129.714

**Published:** 2022-10-25

**Authors:** K Dadaczynski, M Messer, K Rathmann, O Okan

**Affiliations:** 1 Department of Nursing and Health Sciences, Fulda University of Applied Sciences, Fulda, Germany; 2 Nursing Science, Trier University, Trier, Germany; 3 Department of Sport and Health Science, Technical University Munich, Munich, Germany

## Abstract

**Background:**

Digital communication technologies had a crucial role during the COVID-19 pandemic, with the internet and Social Media as highly frequented sources for retrieving health information. University student's health and well-being were highly affected and most interaction with peers and professionals migrated to the digital realm, which made digital health literacy (DHL) a key competence to navigate digital health environments. The main goal of the study was to explore DHL of students in Germany.

**Methods:**

A cross-sectional online survey among students (N = 14916) from 130 universities in Germany was implemented as part of the global Covid-HL Network, collecting data on DHL, physical and mental health, SoC and sociodemographics. Data was analyzed using univariate, bivariate and regression analyses.

**Results:**

Assessing the reliability of information (5964/14,103, 42.3%) and determining commercial interest of information posed the most difficult tasks (5489/14,097, 38.9%). Difficulties were revealed for finding information (4282/14,098, 30.4%). Female students reported lower DHL and social media use was associated with lower judgment skills. 38% of all students reported low and very low well-being and 29% reported at least two health complaints weekly, while health outcomes follow a social gradient (lower SES and gender). Regression analysis showed significant association between SoC and well-being (OR: 1.2-2.03) and health complaints (OR: 1.58-1.71). Higher future worries were with low well-being (OR: 2.83) and multiple health complaints (OR: 2.84).

**Conclusions:**

There is an urgent need to enhance DHL and SoC of students and implement health promotion strategies, using target group specific intervention. Gender and socioeconomic differences must be taken into account and interventions could be delivered within the university. Measures should also address student mental health.

